# COVID-19 related innovation in Aotearoa/New Zealand mental health helplines and telehealth providers – mapping solutions and discussing sustainability from the perspective of service providers

**DOI:** 10.3389/fpsyt.2022.973261

**Published:** 2022-08-30

**Authors:** Alina Pavlova, Bonnie Scarth, Katrina Witt, Sarah Hetrick, Sarah Fortune

**Affiliations:** ^1^Department of Psychological Medicine, Faculty of Medicine and Health Science, University of Auckland, Auckland, New Zealand; ^2^Orygen, Parkville, VIC, Australia; ^3^Centre for Youth Mental Health, The University of Melbourne, Parkville, VIC, Australia; ^4^Department of Social and Community Health, School of Population Health, University of Auckland, Auckland, New Zealand

**Keywords:** telehealth, innovation, COVID-19, helplines, sustainability, mental health

## Abstract

**Background:**

The coronavirus disease 2019 (COVID-19) pandemic and associated interventions resulted in changes in both the demand and supply of mental health services and necessitated agile adaptation and innovation from service providers.

**Aims:**

The aim of this study was to explore what innovative solutions were adopted in response to COVID-19 and the pandemic control measures, what opportunities and challenges were associated with these innovations, as well as to critically reflect on the longer-term sustainability of the innovations in the context of Aotearoa/New Zealand mental healthcare.

**Materials and methods:**

We used thematic analysis to analyse the data from the 23 in-depth interviews with helpline employees and general practitioners from 18 service providers that regularly engage in mental healthcare.

**Results:**

Two key themes related to COVID-19 and the pandemic control measures were identified from respondents’ accounts. These were “Technological innovations” and “Process innovations” where providers noted types of innovative solutions, and opportunities and challenges associated with those. The themes culminated in a meta theme “Sustainability of changes to service delivery” that appeared consistently in each theme and asks to consider how sustainable these innovative solutions might be in the long-term. Namely, sustainability of innovation was questioned in respect to the (a) innovative solutions being the emergency solutions with little or no impact analysis, (b) “returning back to normal” due to limited future funding and innovation as a sunk cost, and (c) sporadic and inconsistent innovation between service providers that does not contribute to quality and continuity of care from the systems perspective.

**Conclusion:**

COVID-19 and the measures of pandemic control were associated with an increase in innovative solutions from service providers. There were both opportunities and challenges associated with these innovative efforts and the sustainability of innovation was questioned. Future research about COVID-19 related innovation of service provision should focus on service user experiences and empirically measure the innovation safety and efficacy.

## Introduction

On March 11, 2020 the Coronavirus disease 2019 (COVID-19) outbreak was declared a global pandemic by the World Health Organisation (WHO). To prevent uncontrolled infection transmission, COVID-19-related deaths and the potential high strain on healthcare services, governments worldwide implemented pandemic control measures. This included measures such as “shelter-in-place” orders, restricted international and national travel, social distancing, and limits on social gatherings. Aotearoa/New Zealand (henceforth Aotearoa) was no exception, and adopted a comparatively strict virus elimination strategy. Summarised by a slogan “Go hard, Go early” (1), the entire country’s population were subject to national and regional strict lockdowns as soon as infections emerged. The lockdowns were characterised by limiting peoples’ movements except for essential purposes such as food shopping, restricted outdoor exercise, acute hospital care and essential work (see Supplementary Appendices 1, 2). Strong protection of international and national borders was also in place.

These pandemic control measures were essential, however, they were also predicted to have impacts on mental health (2), hence a number of mental health interventions were developed to try to mitigate these effects. For example, a range of initiatives under the name *Kia Kaha, Kia Māia, Kia Ora Aotearoa: COVID-19 Psychosocial and Mental Wellbeing Plan* (3) were devised to support population mental health. National mulitmedia campaigns (e.g., “Getting Through Together,” “Struggle Got Real?”) were rolled out to promote mental wellbeing and reduce distress associated with COVID-19, also promoting telehealth services (4, 5). Similar to what was observed overseas (6, 7), in Aotearoa there was a decrease in the use of regular face-to-face primary and secondary healthcare, including mental healthcare and, at the same time, helplines and telehealth providers saw an increase in service demand (8).

There were changes in the nature of mental health helpline and telehealth demand. For example, contact from people of certain demographics (e.g., youth) increased and changes in reasons for contact were also observed (e.g., COVID-19 related anxiety, lockdowns-related concerns). Providers noted more crisis calls and higher complexity of calls from people who had already been engaged in secondary mental health care. Additionally, COVID-19, the associated pandemic control measures, and changes in demand for health services in general also resulted in disruptions in the supply of helpline and telehealth services in mental health care (8). International evidence suggests that the provision of healthcare was affected by workforce shortages, interruptions associated with work-from-home mandates, and greater stress from operating during such unprecedented times (9–12).

The changes in both the demand and supply of health services necessitated agile adaptation from the service providers, including in mental health. International studies have shown that the COVID-19 pandemic may have a silver lining by inspiring innovation that might be beneficial to healthcare services in the years to come. Examples of such innovations are many, ranging from electronic referrals to greater adaptation of telehealth to digital home-monitoring for people with long-term conditions (13). Additionally, innovations that arose as a result of COVID-19 were characterised by significantly more collaborations across multiple providers and predominantly occurred in digital spaces (13, 14). More funding without stringent reporting criteria was available, which encouraged greater creativity (14); “frugal innovation” (i.e., doing more with less) was also commended (15).

Along with the favourable long-term impacts of creativity and innovation, potential challenges were also noted. Telehealth, for example, may challenge therapeutic relationships between clinicians and service users, lacking the usual non-verbal cues and as well as the lack of established “webside” manner (16). Moreover, due to the speed of innovation, thorough impact analysis or research that demonstrates safety and efficacy of new solutions was often lacking (14). Empirical evidence is needed on the experiences of mental health service providers and service users who are utilising the innovations.

There is still little known about the innovations adopted by helplines and other mental health services in Aotearoa in response to operating under COVID-19 related restrictions.

The aims of this study, therefore, were to focus on Aotearoa helplines and telehealth service providers by exploring what innovative solutions were adopted in response to COVID-19 and the pandemic control measures, what were the opportunities and challenges associated with these innovations, as well as sustainability of these going forward.

## Materials and methods

This study is a part of a broader study that investigated the use of helplines and telehealth support in Aotearoa during COVID-19 pandemic control measures that used a mixed-methods research design (8). It was approved by the University of Auckland Health Research Ethics Committee (AH3109).

This current study uses a qualitative thematic analysis approach following Consolidated Criteria for Reporting Qualitative Research (COREQ) guidelines (17).

### Participant selection and sampling of organisations

Between 1 November 2020 and 30 April 2021 we purposefully sampled (by funding type, size, areas of expertise, target population age, gender, ethnicity), and approached 23 (of approximately 33 that exist) national helplines and support organisations (henceforth commonly referred to as helplines) and 13 General Practices across Aotearoa (selected by area and practice size).

**FIGURE 1:**
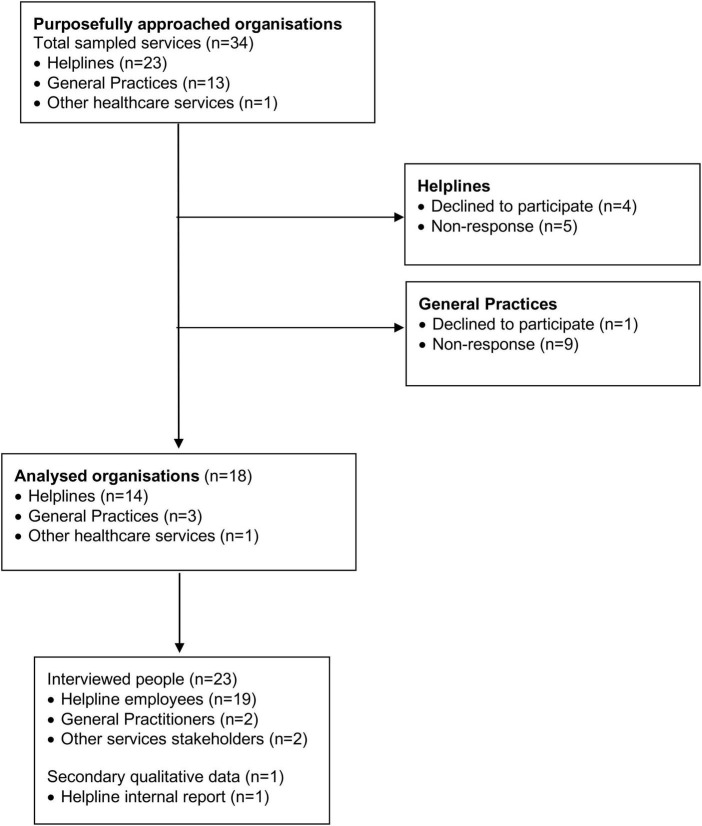
Data collection flow diagram.

Fourteen national helplines, three General Practices, and one other healthcare related organisation agreed to participate in qualitative interviews (see Figure 1). The fourteen national helplines represented those with public and private funding, a range of sizes (8-350 + staff/volunteers), different areas of expertise (general health, general support, general mental health, specialised services targeting presentations of anxiety, depression, substance use, eating disorders, suicide and self-harm support etc.), and various target populations such as age (general, services for youth, services for elderly), gender and sexuality (general, specific to male, specific to female, LGBTQI +), and ethnicity (general, Māori, Pacific Peoples, Asian populations, people with refugee status). Some of the organisations were umbrella organisations representing multiple services. All of the General Practices were urban based, two in the largest metropolitan centre – Auckland, Aotearoa. We also included interviews with participants from one other healthcare related organisation to gain insight into the contextual issues from a broader perspective.

We used a combination of snowball sampling and directly approached organisational gatekeepers. We found the relevant gatekeepers’ names and email addresses via organisational websites and/or professional social networks (i.e., LinkedIn) and asked them to distribute information about the study among staff/volunteers. Two-fifths of the final participants were recruited by direct approach, with the remaining three-fifths being recruited by snowball sampling. Four of the helplines and two General Practices declined to participate, and five helplines and nine General Practices never replied. Reasons for not participating were not explained, but participating organisations described difficulties in finding time for the research participation due to increase service demands.

### Procedures

The Chief Executive Officers (CEOs) of organisations were approached for consent to participate. Once consent was obtained, we then approached potential key informants, explaining the methodology and study aims, and sought consent from key informants directly. In the case of non-response, we followed up with the organisation once every 2 weeks to a maximum of four times. Most participants preferred for their interviews to take place on Zoom (conducted in private settings where the interview could not be overheard). Two interviews were face-to-face, and two participants replied in writing. In addition, one organisation shared qualitative data available from their own internal review.

We used a semi-structured interview guide (see Supplementary Appendix 3) to elicit open-ended responses relevant to our research question (18). The interviewer did not previously know the participants with one exception. Interviews were audio-recorded and transcribed by a professional transcriber under a confidentiality agreement. Transcripts were de-identified by the interviewer. The audio recordings and identifiable parts of interview transcriptions were permanently deleted.

### Research team and reflexivity

The research team consisted of five academic researchers (including researchers with lived-experience of mental health recovery) of diverse ethnicity, currently residing in Aotearoa and Australia. Some researchers were trained clinicians and many held additional clinical, suicide prevention, or other advisory roles. All members of the research team identified as female. The project was funded by the Oakley Mental Health Foundation.

### Analysis

An atheoretical thematic approach was used to analyse the primary data (19, 20). Namely, we used a general inductive process (20) building from a data up while being guided by pre-established research questions. Two researchers conducted independent coding and two other members of the team looked at the data to minimise the risk of idiosyncratic interpretation before themes were discussed with the wider research group. We kept written memos with reflections on how personal beliefs and values could affect interpretation of the data, keeping in mind our aim to contribute to the improvement of national helpline services and telehealth as used in the context of primary care.

The results were analysed in the context of Aotearoa COVID-19 pandemic related general (Supplementary Appendix 2A) and healthcare (Supplementary Appendix 2B) restrictions associated with the official Alert Levels (see Supplementary Appendix 1).

## Results

### Participants characteristics

Between September 2020 and March 2021, we interviewed 23 employees/volunteers from the included orgnaisations. Study participants mostly held managerial positions; 35% of participants were in direct contact with service users (Table 1).

**TABLE 1:** Qualitative participants characteristics (*N* = 23).

Characteristic		Count
Gender:	Male	7
	Female	16
Age:	25–64	22
	65+	1
Ethnicity*:	NZ European/Pākehā	15
	Māori	2
	Pacific Peoples	2
	Asian	3
	Other	1
Seniority*:	Managerial role	18
	Non-managerial role	8

*Not mutually exclusive.

### Summary of themes

Respondents reported that COVID-19 and the associated pandemic control measures, as well as continuous media reporting, were a “shock to the system” that necessitated innovative solutions to keep the services going. Respondents also noted that this shock to the system was an opportunity to innovate and work differently, especially as a new stream of the COVID-19 related funding became available.

Two key themes relating to these changes were identified from respondents’ accounts – “Technological innovations” and “Process innovations” – where providers noted types of innovative solutions, as well as associated opportunities and challenges. The themes culminated in a meta theme “Sustainability of changes to service delivery” that appeared consistently in each theme and noted the need to consider how sustainable these innovative solutions might be in the future and what questions we should ask (Table 2).

**TABLE 2:** Themes and sub-themes for qualitative data analysis.

Meta-theme	Meta-theme outline	Theme	Theme outline	Sub-themes
*Sustainability of changes to service delivery*	*Rapid crisis-related innovations may not be sustainable:* (1) *there is a risk of going back to “normal” when the situation improves and/or as the funding is withdrawn*, (2) *there is a need for impact analysis of what works and what does not for innovations that have not been tested*, (3) *another key sustainability question should be focused on whether innovations be sustainable in a system that is not.*	*Technological innovations* *Process innovations*	COVID-19 related changes have sped up technological innovation in service delivery, promotion, and how organisations were run. COVID-19 related changes in demand volume and nature necessitated process innovation such as introduction of new services and working together.	• *Types of innovations (i.e., telehealth, remote working, smart promotion and IT infrastructure)* • *Positive impacts of technological innovations* • *Challenges associated with technological innovations* • *New services* • *Proactive approaches in service promotion* • *Working together (inter- and intra-organisation and with service users)*

### Theme 1: Technological innovations

#### Type of technological innovations

Participants noted that COVID-19 and associated pandemic control measures sped up technological innovations, including further adaptation to novel telehealth solutions (i.e., via Zoom or DoctorSeeMe) to engage with service users, e-referrals^1^ and e-prescribing,^2^ remote working solutions to facilitate organisational processes remotely (i.e., online chat apps such as Slack, Zoom, and Microsoft Teams), online telephony (e.g., Amazon Connect Services), online cloud platforms (e.g., PureCloud), and associated improvements of IT systems.

##### Innovation in service delivery

Depending on the organisation, technological innovations varied in scale. For some organisations innovation entailed easy solutions such as switching from face-to-face to telephone calls. Other organisations explored more creative solutions. For example, one respondent described an innovation in the area of family violence support that “*created a hidden web chat for women who couldn’t call [*…*] because their abusive partners were in the room” (Participant 16).* Another organisation mentioned creating online psychoeducation programmes increasing access to families:

*“Parenting programmes were delivered on Viber, all youth programmes were online via TikTok, Viber, WhatsApp, Facebook and Instagram. Cross-Cultural Facilitators set up community e-groups using WhatsApp and Viber to talk to families and leave audio messages in multiple languages”* (*Participant 22*).

Finally, hybrid interventions using multimedia approaches were mentioned such as providing audio and video materials for breathing exercises so during the call “*[service users could] watch the video and practice (it) together. Or download it and do it later*… *so it becomes like a multisensory sort of intervention where you’re not just talking to somebody on the phone that you can’t see*” (*Participant 10*).

##### Remote work: Enhanced recruitment and employee retention

Other than new and enhanced services, technological innovations enabled a more diverse workforce to be hired. As people started to work from home the possibility of remote hiring became a possibility:

“*It has strengthened (us) and we’ve grown the service in terms of representation, diversity and inclusion. We can include people who have got disabilities and they’re not able to come along to in-person training. Or they might be rural, so that is a huge thing.” (Participant 10)*

Remote hiring also allowed qualified New Zealanders working from abroad to be hired as well as “*people with more clinical experience or health backgrounds than* (…*) we get in Auckland.*” (2). Finally, it helped in terms of retention of staff who were relocating but maybe wanted to stay with the job:

“*We had a volunteer who came back, he’d left in March because he went to a different University and he came back onboard. He was like: “Oh gosh you guys have nationwide volunteers I wanna come back.” And so it’s really cool now that not only can we keep the volunteers if they move*.” (*Participant 13)*

##### Technological innovations in service promotion

Technological innovations were utilised in service promotion. One provider highlighted their approach via geotargeting: “*So with peer support we timed it, we did the age, the time of day (and these are the services available from 2 to 10 pm) and we usually do a lot of promotion after hours so had to be smart about it targeting high deprivation at 9 and 10 areas”(Participant 19*). As well as mixed-media digital/non-digital promotion advertisements: “*so what was really cool we did digital billboard so if you pass this billboard and you’ve got Bluetooth on, when you next go online you’ll be given, served up that ad. So that could get us into regional populations as well*.” (*Participant 19).* Likewise, technology was incorporated to use more appropriate channels for targeting cultural minorities that could be otherwise difficult to connect with. For example, for Chinese service users to promote helpline services some providers used “*WeChat platform that many Chinese connect with*” (*Participant 18*) with great success.

#### Positive impacts of technological innovations

According to providers, these technological innovations had many positive impacts for service users and employees/volunteers delivering the service.

##### Flexibility, individual approach, and outreach

Providers observed that service users “*enjoyed not having to travel to their appointments, not having to take time off work. So it was a lot more convenient and time effective” (Participant 5).* This was similarly true for using online prescriptions where “(*Patients) didn’t need to drive past the surgery picking up the scripts.” (Participant 3).* As one provider emphasised, for youth especially *“connecting online (was) a preferred way for them to communicate.” (Participant 22).* Digital connectivity also enabled service providers to reach out and expand service delivery to previously underserved populations.

##### Enriched practice

Technological innovations provided new opportunities that enriched practice. As a doctor explained “*talking to the patient (while) drinking your cup of coffee at the same time*… *made it more a casual (and) made our patients talk to us more or better.” (Participant 3).* The same doctor noted how family participation helped to enhance consultations:

*“The daughter who knows me, and is a patient as well, walked past and just spoke into the camera. She said, Doctor, she’s not talking to you about her leg which is swollen, you know, like dobbing her in? The mother was like, go away, this is my consultation. So in a way, it was quite funny having the whānau, the support there, helping enrich the history that I got.” (Participant 3)*

Being in a client’s home also provided some new intervention opportunities:

“*So I’m in the client’s home [*…*] I’m in the place where they’ve perhaps had panic attacks so let’s look at that. We could walk around the house with them, they could take you into places where they might get anxious, practice exercises with them in situ. This is an incredible opportunity.*” (*Participant 10)*

##### Improved organisational practices

From the perspective of providers, technological innovations allowed for staff to be able to work from home, safe from infection: “*a really quick bit of innovating [*…*] made a big difference because our team [*…*] felt safer. Their anxiety dropped. They could be in their bubbles with their family*” (*Participant 9).* It was also helpful for staff who had other reasons to remain home, such as family commitments.

Some respondents commended remote/online training and opportunities for more realistic helpline experience:

“…*when (volunteers) were doing the helpline practice call they would turn off their screen and just have the headphones. And it was literally mimicking a helpline call and so they got a super legit practice experience. And with Zoom it’s amazing you kind of sit in the room and watch them but you’re not intimidating them, you turn off the screen and they almost forget you’re there*” (*Participant 13)*

Additionally, in organisations delivering their service nationally, colleagues were able to meet remotely for the first time “*they got to talk to other volunteers all-round the country as well and to see them on Zoom it was really cool” (Participant 6).* Managers spoke about improved efficiency “*We’ve actually created a situation where our communication between helpline counsellors and triage staff and managers has actually sped up considerably.” (Participant 10*)

Finally, solving hardware problems that could potentially result in savings was also noted:

“*We didn’t have enough kits to be able to suddenly give everyone a whole kit to work from home. And so (the IT) created this fantastic virtual desktop which meant that you could use your own device at home and log in and do your work” (Participant 9)*

#### Challenges associated with technological innovations

Even with considerable positive effects of innovation, providers highlighted multiple challenges.

##### Accessibility

With regards to the technological innovations, providers noted variability in access and potential for exacerbating inequities:

*“(Some) people that didn’t have the technology, didn’t have the strength of the internet to be able to do that. And that discriminates those people. (In such cases) we had to default to the phone which just means you lose out on all those visual cues and I worried that I wasn’t going to service them as well as I could if I was seeing.” (Participant 3).*

“*Some older people, things like technology, it wasn’t so easy for them” (Participant 13).*

Providers also mentioned that young people or people in rural communities might not have had enough mobile telephone or internet data or a device to use. Likewise, some service providers including staff and volunteers also had less access to hardware:

“*These (new) systems require really good computers, you know, when you’re running Microsoft 10’s to connect the help, to connect all our triage and helpline customers, you’re running PureCloud, you’re running all these online systems. We found for a lot of volunteers and out staff it was “chewing up” their computers.” (Participant 1)*

##### Acceptability

Even when people had the technology, it did not work for everyone. Participants observed that telehealth was difficult for young children and, in certain situations, it challenged the assessment *(“I can’t assess my child who’s got terrible epilepsy by Zoom, you know*?”(*Participant 3)).* There were concerns noted about providing support to those who “*had thoughts of self-harm or suicide, (or were) sexually assaulted*…*not being able to connect with patients and have that rapport, particularly when you’re talking about safety planning and things like that. (It) can be quite challenging. (Participant 17*)

##### Privacy and safety

Privacy concerns were also noted:

“*Parents (were) protecting their children from their conversation, so they won’t talk about (what they needed to talk about), with our volunteers while their children are around. So yeah, the opportunity to talk to them was less.*” (*Participant 6)*

Providers also had to take responsibility for service users’ privacy:

*“We had to be really specific about what we would accept when (soliciting medical images). It had to be anonymous, there had to be no identifying feature, so certainly not a face in the shot. You took a picture of the area itself. So, it’s all of that – safety, security, privacy. The IT guys were working red hot hours (to meet) the kind of quality, privacy and ethical standards of health care” (Participant 7)*

The transition to working from home did not allow service providers to hold the callers in the same way:

“…*we are holding this person who is high risk, who can we share the responsibility with? You get people who are suicidal or wanna harm themselves*…*over the phone*…*it’s very challenging [*…*] because you’re working from your home. your colleagues are not there, you don’t have your line manager to debrief with. to validate that you’re doing okay*…*there’s also. lack of resources as well when you’re working distantly.” (Participant 20)*

With regards to quick online onboarding, providers worried about service users’ safety due to quick hiring without sufficient training:

(“*(new online councillors) were connecting with our tangata whai ora (consumers) to help us with volume, but they’re not going to get two weeks training induction. They’re not going to get all of the things that a normal team member coming into the organisation was going to get”. (Participant 18)*

##### Efficiency

From the providers’ perspective, working distantly sometimes hindered efficiency. In terms of consultations, missing non-verbal cues made consultations last longer:

“*[Counsellors] were having a real problem and the sessions and were running over time because what was happening is they’re doing their Zoom sessions and they [.] communicate with their hands, you know, how they’re ending the session. [.] So the clients are not able to get that special effect, so they were [.] finding it a bit harder ’cause of the camera and the types of cues that they use and to help the client understand that the session has ended” (Participant 10)*

##### Impact on service providers

Additionally, without the usual office routine, workers noted that they found themselves working overtime more often, missing collegiality and support, which negatively impacted their own mental health: “*I’ve sat in the morning and I’ve looked up and it’s three o’clock and. I don’t know how those hours have passed, you’re just on the phone or you’re triaging*…*it comes with a lot of responsibility working from home, because you wanna make sure that you’ve done the best that you could for each caller*…*but that’s at the expense of your wellbeing” (Participant 8*)

Boundaries were vital for organisations navigating ethics and working from home. However, being in one’s personal space often made it difficult to enforce those boundaries and put further pressure on staff to maintain a safe, confidential, professional working environment, while managing day-to-day home issues. The necessary boundaries of working from home made it a more isolating experience compared to being in the office with colleagues who are bound by the same confidentiality.

“…*so privacy for at home*…*so if I was briefing a volunteer*…*if their husband or partner was home, they had to work and find that privacy. And for me it was the same, if my (partner) was home [*…*] we had to be private. And so we would have to isolate ourselves in different parts of the house, which was tricky because you wanna be where you’re comfortable*…*But the conversations about our job*…*you’re talking about some pretty gnarly stuff.” (Participant 15)*

Many unpaid staff that helplines often rely on were not available for remote work:

“*70% of our volunteers didn’t want to take calls from home*…*And because of the nature of volunteering* (…) *we don’t pay these people, they simply don’t turn up.” (Participant 1)*

Challenges in terms of training and onboarding new staff included being impersonal, especially when there was a big group, and when the trainees preferred their cameras to be off.

“*Trying to recruit people virtually 100% is probably the most challenging thing. It’s quite difficult to recruit, onboard, and train a person fully virtual (and that was) the most challenging part of our journey.” (Participant 14)*

### Theme 2: Process innovations

#### New services

Innovation was not limited to technology. Providers noted that COVID-19 and associated pandemic control measures resulted in new services and approaches being developed to address changes in demand. New helplines targeting a specific population (e.g., Pacific Peoples) or specific types of presentations (e.g., family violence) were initiated. Peer support services were implemented. In addition, existing services expanded their activities to include the delivery of essential items to service users (e.g., food, personal protective equipment (PPE), paying utility bills):

“*We were providing a heap of food parcels. Our teams were going out delivering food parcels to families, blankets – during that time when we’re having to provide extra [*…*] to best support families*.” (Participant 4)

Some of the new services were introduced due to changes in demand and according to populations’ needs:

“*Safety, basic needs become priority for (a) person [*…*] So it just, calling them and saying, “Oh are you okay?” And they need food*…” (Participant 18)

*“They open (the COVID-19 information letter) and the information is all in English [*…*] we knew that wouldn’t be effective. Even when it came in English, they didn’t understand it, you know [*…*] So we set up and orientated a Pacific helpline”* (Participant 7)

Other services (e.g., family violence-related services), were initiated based on international evidence indicating this area being problematic and the need to be prepared, even though not all fears were realised:

“*The need for (support with family violence) we already knew from women so Italy – the first country in the world apart from China to go into lockdown. And that’s where the first reports were about this increase in family violence. You would expect that you know, people being locked in a house. But it was very interested to see*… *I was expecting that we would be awash with phone calls, (but we didn’t)”* (Participant 6)

Peer support was established in anticipation and as a response to the increase in demand:

*”That really was an innovation that came about specifically as a result of the need to respond to community mental health, the stress, anxiety as a result of COVID”* (Participant 19)

#### Proactive approaches in service promotion

In anticipation of COVID-19 related mental health presentation based on what was observed internationally, respondents emphasised a more proactive approach to contacting service users – “*We proactively talked to our older patients, the nurses and receptionist, just rang*” (*Participant 3*). They also described how their organisations expanded their services by adding proactive health communication (i.e., newsletters, frequently asked questions, how to connect to your community). An example of feedback about proactive check-in calls, was in the context of staff who were highly opposed to it due to fears of it being *“(re)-traumatising (because) they’ve got lockdown and (we are) still ringing about the suicide a year ago*…*But in effect it, they were so grateful that we’d rung them, that we’d thought of them. So it was really a good social survey.*” (*Participant 6*)

The innovative approaches were also noted in helpline services promotion in a broader sense, for example, describing the nature of the service, especially for people who might be experiencing distress for the first time but had never called a helpline before (e.g., “*we try and explain what happens when you text or call a mental health helpline” (Participant 19)*) or find it stigmatising (e.g., “*[in many cultures seeking mental health support] comes with it’s own set of values and beliefs things should be kept in the house, don’t talk about*…*.So we sort of addressed the issues of confidentiality and tried to tell them coming to counselling is important, what is counselling? It’s definitely not akin to them being crazy.*” (*Participant 18*)). Helplines advertisements also targeted whānau (family), for example, services that targeted males “*encouraged partners as well to contact us as a safety plan for men” (Participant 17*). Many services used a saturation method of targeted advertising to ensure that key populations were reached early on and used high messaging repetition. To find out what was important for service users, providers actively engaged with them.

#### Working together

##### Working together with service users

In addition to increased staff collegiality, or as one participant described – a feeling of “*being part of the same waka (boat*)” (*Participant 17*), many (new) services and promotion activities have also increasingly relied on working together and co-design. As noted above, organisations were progressively working together with service users and co-designed services that aspired to address the community needs. Thanks to co-design, respondents noted that “*mental health and wellbeing campaign(s*)…*(were) culturally and linguistically appropriate and accessible for communities from (diverse) backgrounds*.” (*Participant 24*). For example, advertisements used Te Reo (Māori – Indigenous Peoples of New Zealand – language) to “*improve contacts from Māori*” (*Participant 19*), used colloquialisms, “kiwi-isms” (e.g., “Grotty Year Got You Shook?”) and bold fonts that were said to better grab attention.

Doing co-design not only helped to understand how to engage with people, but also what services were needed. For instance, “*overwhelmingly (Māori) people said [peer-support] is a good model, I would feel comfortable talking with someone who has lived experience of the kinds of things that I want to talk about. I haven’t been to a counsellor before, don’t worry about the counselling service. We used some smart gains from the co-design work to help us make sure that the service was designed well and mainly focused on Māori*.” (*Participant 19*)

Co-design was seen as successful and associated with more targeted promotion and services, seeing “*an increase in access (as) a good thing, not a bad thing*” (*Participant 5*), indicating that co-design has helped to reach people and normalised help-seeking. Additionally, instead of high spending trying to reach everybody, engaging community in co-design and promotion meant better reach at lower cost.

*“It is really using smart ways – using small budget, and using it well [*…*] (instead of using an) advertising agency or media agency, that we actually put on promotion – we did really good geotargeting, [*…*] we timed it, we did the age, the time of day, placements” (Participant 19)*

##### Partnering and outsourcing

Service promotion activities also relied on “*activating partners (such as) Mental Health Foundation, talking with police to share these messages, Salvation Army, Plunket, primary care, social media influencers*” and cultural leaders and organisations (e.g., Pacific churches). Partners were often given additional assets such as posters, t-shirts and other media resources to help in spreading the message. As summarised by one participant:

“*There are some really good provider groups that work together, where there are points of connection where we can share resources and discuss things.*” (*Participant 10)*

Hence, asking for help was as important as helping partners who requested assistance, or proactively trying to “*to understand whether there was a particular need [*…*] to provide some additional support to increase the awareness of helplines in (certain) regions, which has been significantly affected by the socio-economic impact of COVID.*” (*Participant 19).*

Besides engaging the service users and collaborating with partners with regards to service promotion, other inter-organisational initiatives and smart outsourcing were noted by the interviewees. For instance, one provider used a company to set up and manage all the telehealth licences, setting service users contact details up in the system and managing all of the bookings. Another company contracted a specialist peer support service instead of having to set up a new service themselves. Some organisations mentioned taking advantage of the new technology and remote working and getting subject expert speakers to talk to the team and give educational workshops – something that has not been done before. External collaborations also meant that essentials such as food parcels and PPE could be delivered. Moreover, providers noted some of these collaborative strategies, such as outsourcings certain activities (managing software packages, finding and training peer-support workforce) worked well in terms of time and cost saving. As one provider summarised:

“*Money wouldn’t even be something that I’d even be thinking of [*…*] (things were done) more in the way of bartering” (Participant 17)*

### Meta-theme: Sustainability of changes to service delivery

Considering the technological and process innovations that emerged because of COVID-19 and the related opportunities and challenges, a meta-theme of sustainability became pronounced during the data analysis. Three aspects of sustainability noted by the providers were particularly salient. Namely, (a) the return to normal and discontinuation of useful innovations, (b) quick innovation without a thorough impact analysis that may perpetuate inequities in care, and (c) variability of innovation between services that can make it difficult to operate as a coherent system.

#### Return to normal

Participants described a tendency for organisations to ‘return to normal’:

“*I have to tell you that the evidence suggests that the district health boards (DHBs) are returning to business as usual very, very quickly.*” (*Participant 16)*

In addition to such path-dependence, funding was also inextricably linked to this theme. Respondents were grateful that the government helped with additional funding that was necessary to help services continue operating during COVID-19 restrictions. However, there was a fear that the funding is finite and they will not be able to maintain new services, even those that were successful (“*(Whether the innovation is going to stay) is not decided at this point* […*] all of the Covid funding was one off funding, it wasn’t ongoing funding.” (Participant 2)*). Some providers noted that due to depletion of funding in combination with continued increased demand they might not even be able to maintain standard of care. Especially because funding applications take time that the organisations do not have:

*“(The services) are absolutely stretched to breaking point [*…*] The time that it takes to write applications and then to report. It’s time we haven’t got.” (Participant 5)*

#### Quick innovations without impact analysis

There were concerns raised in relation to the challenges outlined in each theme, such as accessibility and acceptability of innovation for service users and impact of innovation on staff.

Although not explicitly voiced, some providers prided themselves on quick solutions without considering the consequences. One provider’s quote exemplifies the ease of transition. However, it does not consider the issues described above – in this particular instance – ensuring the safety of both service user and the carer:

“…*if you’re expanding things rapidly the biggest challenge in terms of telehealth was getting the personnel on board. One of the things was to utilise peer workforce and that happened relatively smoothly and relatively quickly” (Participant 2)*

Indeed, rapid changes without a thorough impact analysis, even those that brought new efficiencies, often resulted in such negative consequences such as high turnaround, staff being overstretched, indicating that tele-mental health would require more thought in the long term:

*“(The amount of volunteers who left because the could not work from home) was quite high, it put an enormous amount of pressure on our triage team. (It) was not that their space wasn’t right*…*they weren’t capable. (It was hard to support) other people while they’re trying to support themselves and their whānau or family in their bubbles [*…*] the presentation to us was “I can’t support myself, there’s no way I’m safe to sort someone else” [*…*] We end(ed) up with a situation*…*that there were a fewer pool of workers doing quite long hours” (Participant 10)*

Moreover, providers found it difficult to find support and direction on how to manage this situation from governing bodies, although they were actively looking for it:

*“I reached out to the New Zealand Psych Board and said give us advice*…*there was no platform anyone was recommending. They still haven’t responded to my enquiry. I sent an email, I even tried calling them, no response, nothing on their website*…*we’ve got a team of psychologists. They all want to keep their practicing licences. They need advice on what is okay to use for tele-health services, ‘cause we’ve never done it before. what’s a safe way to do it and so we just had to work out what systems are available. there was a lot of pressure*…*just operate at 100%” (Participant 19)*

Evidently, many organisations acted fast to the best of their abilities but the suitability and sustainability of these rapid innovations will ultimately have to be reviewed to make them future-proof.

#### Can innovation be sustainable in a system that is not?

Finally, providers emphasised that sustainability of innovation can be hindered by not being embedded in a coherent system. As we noted above, many of the smaller non-for-profit organisations showed agility, but also found themselves spending more time to piecemeal contracts and grants that required increasingly time-consuming processes to obtain and maintain. Conversely, bigger organisations often had the infrastructure and investments, but less welcoming attitudes toward innovative efforts:

*“So we have a significant portion of our workforce who are not digital native, you know, there’s even a proportion of them who don’t use email. So we know that there are some attitudes amongst the staff themselves that will impede the take-up of digital consultations.*” (*Participant 5).*

There were discrepancies between the level of innovation between services (e.g., “*some GP practices simply didn’t have the infrastructure to have virtual consults. And then some of the specialist mental health did have the infrastructure to support virtual consults*” (*Participant 5*). These incongruences complicated how well the system can work as a whole. Providers described that even if their services strived for improvement, the efficiency was not guaranteed as they ultimately had to rely on adjacent services that may not have functioned well:

*“We are holding this person who is high risk, (but) who can we share the responsibility with? Because we are only a voice at one end. Holding a client and trying to stop them from harming themselves then (having no one that we can) share the responsibility (with). It’s tough!*” (*Participant 18)*

Similar sentiment was echoed with regards to innovative health promotion that showed good results. Yet, many helplines did not have the capacity to deal with the surge in demand. As one provider noted “*every time (there was a health promotion), we get more calls and then we have to stick our hand in our pocket to pay for it, so… (laughing)” (Participant 19).* This was exacerbated by working at capacity during a global pandemic where services were increasingly working outside their scope with more complex presentations, over-relying on volunteers, and new staff were hired and services introduced with less-than-usual oversight and implementation processes.

Many noted that there is a greater need to look at long-term innovative solutions in terms of *how* the services currently operate across the health system:

“*By the time (service users) ring the helpline in distress [*…*] we’re actually the ambulance at the bottom of the cliff, right? And I say look, [*…*] if I use the same analogy my counsellors are sort of halfway down the cliff grabbing people as they’re falling. Not getting on top of this in front of this means we’ve got to stop people falling off the top of the cliff [*…*] So I think the solution is not the our regional or National helpline, the system’s gotta be a government, a community, you know, academic response to what the hell is happening. And how do we get on top of it for a change rather than just keep throwing money at it and backwards, you know, from behind [*…*]” (Participant 1)*

Providers noted that COVID-19 related crises elucidated and exacerbated issues of sustainability that were already there with regards to healthcare system at large. Some tangible solutions were also proposed:

*“What I find frustrating, especially with mental health, [.] there’s too many barriers still [*…*] The funding for primary mental health which has come through, allows us to then refer them to either a psychologist or a counsellor [*…*] My nurses and I know these patients well. If we say they are in need of help for their mental health […] we’re serious, we’re not making it up. And if we can handle it, we will do it, but sometimes [*…*] they are really in need. But they somehow don’t meet the scores, the criteria. And so I feel the criteria’s a bit too blunt and it just acts as another barrier [*…*] (It would be more helpful) if I can be funded to see them more or to see them for an extended consultation, because I’ve already built that relationship with them [*…*] Or fund my nurses so they’re able to follow-up with them [*…*] rather than just handing them on to another provider where they have to start all over again [*…*] I’m really happy for increased mental health funding, but I think we, as in the health system, could’ve been smarter at how it was disseminated*.” (*Participant 3)*

## Discussion

After the New Zealand government announced level 4 lockdown, which required all but the most essential workers to work from home, services had 48 hours to adapt to working remotely. This put enormous pressure on mental health services, and the pressure continued throughout lockdown as services rapidly learned and adapted to the new working environments. Similar to what has been observed internationally (13, 14), a great deal of creativity and innovation emerged out of this necessity to adapt. Providers’ adaptability ranged from implementing highly technological telehealth and remote working solutions to increased cross-functional collaborations (13, 14). In addition, in Aotearoa, providers sought and utilised creative health promotion solutions utilising new tools such as geotargeting and multisensory advertisements and heavily relying on co-design. The agility of adjusting one’s business model and temporary providing non-core services that are of greater need (e.g., delivery of essentials, proactive checking-in) was also noted.

Many of these innovations resulted in service improvements with service providers commenting that both staff and service users appreciated additional flexibility from remote working and telehealth. Accessibility improvements were noted with service users who were previously not able to access services due to living remotely and being short on time, were now able to access services online. Remote working technology helped the organisations to diversify the workforce, which could have positive flow-on effects in addressing specific populations’ needs. Working with clients online provided additional therapeutic opportunities and, for some, helped to build rapport by being in a more informal setting. Finally, some innovative solutions, such as utilising inter-organisational bartering, increased service efficiencies which resulted in cost savings without compromising service delivery – a novel solution from Aotearoa that could be utilised more widely internationally.

From a different perspective, providers emphasised that many innovative solutions represented a quick fix and some of those might not represent a long-term solution. As prior reports have suggested, speedy innovation is often lacking impact analysis but should be considered at a later stage if innovations are to stay (14). Although accessibility of services was increased for some, switching to one hundred percent telehealth mode illuminated inequities – not everybody had equal access to internet, data, or even hardware; not everybody – both service users and staff – had a safe, confidential, and professional environment for an online consultation, similar to what has been reported in other studies (23, 24). Telehealth varied in effectiveness depending on types of presentation. This report established what types of presentations were less suitable for telehealth with providers noting that it was difficult to assess physical presentations, engage with young children, and read the non-verbal cues from patients with more severe mental health concerns. Hence, hybrid or individualised solutions might be more suitable with regard to telehealth.

In line with international reports (9–12), for staff, working from home made it a more isolating experience compared to being in the office with colleagues who are bound by the same confidentiality and could provide support. Staff were not held through the turbulent times and the question that remained unanswered was “who cares for the carers?”. The change in working environment might be among the reasons that can explain the reports of a decline in psychological well-being of healthcare workforce during the COVID-19 pandemic (25). Consequently, many providers noted that while innovation was needed and some of it has proven to be effective, as we are settling in to a “new normal” we need a more nuanced discussion about what works and what does not, and what improvements can be made. Prior qualitative evidence echoes these observations with mental health workforce embracing telehealth because of its scheduling flexibility and increase in attendance from service users, but noting potential inequities for service users and potential negative effects on one’s job satisfaction and wellbeing (23). Our results contribute to the literature emphasising the need to consider hybrid solutions between face-to-face care and telehealth and more flexible working environments where online work is a choice rather than a necessity. Henceforth, considering innovation pros and cons and thinking about the future, it is important to focus on the sustainability of innovation.

As pointed out by other authors (14), evidence-based research is needed to establish the innovations’ safety and efficacy. Moreover, there is a need to consider how hybrid models of care can work in the future and to develop new clinical codes and protocols for working in digital environments, what one study has described as a “webside” manner (16). Learning from success in other jurisdictions, and when safety and efficacy are established, innovation in Aotearoa could also leap forward to more cutting-edge innovations in collaboration with research institutions, such as applying artificial intelligence (AI) to identify new treatment pathways (26) or utilising chatbots for more effective triage (27) that sprang out in other counties but that we did not see mentioned in our data.

Coming back to the matter of sustainability, another issue that seems apparent from analysing providers’ accounts in our data, but not currently echoed in other studies internationally, is that there is a risk of the innovative efforts only being implemented short-term and innovations being abandoned once COVID-19 funding dries out. Some of the innovations are funded temporarily and already have an end-date and will not be continued without additional funding. Although not explicitly mentioned by providers, it is also likely that innovations that require continuous spending (e.g., software and digital subscription technology such as Cloud computing) will be let go without additional funding. Consequently, research is needed for the organisations to advocate for funding of innovative solutions that proved effective.

Not all the innovation, however, comes at a cost. Our data has shown that the most cost-effective solutions came from collaborative efforts and working together. Co-design, outsourcing and bartering have shown to be effective strategies with Aotearoa providers, especially with regards to outreach. Hence, frugal innovation was also noted and praised by the providers (15).

Last but not least, as it was brought forward by the providers, even with innovative solutions being in place, the increased demand could not be maintained sustainably without a more systemic healthcare change. In other words, participants noted that innovation was not a panacea in a system where there are differences in services agility and ability to serve the increasing demand. Organisations do not work in isolation and even if a helpline or a General Practitioner have state-of-the-art systems, when patients are referred to other services, they might not be able to get the help they need. In accordance with prior evidence (24), it was noted that larger organisations were better equipped to adopt telehealth. In contrast, smaller organisations were more agile and able to innovate, creating an even greater gap in how the services are delivered.

As such, collectively, it seems that we have not learned from previous pandemics (28) about the need to be prepared (e.g., via surveillance systems), to have a surge capacity, and to maintain a healthy healthcare system in general to be able to respond to crises and optimise emerging technologies and innovations (29, 30). There is still a need for more strategic and innovative thinking in how the healthcare system runs as a cohesive whole based on suggestions from this and prior reports (31).

## Limitations and avenues for future research

This study should be considered in light of some limitations. First, although we interviewed service providers and know from other studies that service users’ satisfaction with services builds on the ability of the provider to build rapport and seamless technological connection (e.g., non-interrupted calls or other technological limitations) (32), we still do not know whether service providers experience reported in this study would reflect the experiences of service users. Therefore, future research could validate the findings of this study by looking more closely at service users’ experiences qualitatively, insomuch as quantitatively. Moreover, although most of respondents had direct contact with service users, they also mostly held managerial positions. A follow-up the results of this study could be triangulated by conducting studies with first responders (e.g., volunteers, councillors, nurses). We also acknowledge a lack of rural perspective in this report. Finally, this is a qualitative study about a range of innovations. A deeper understanding of innovations is needed to ascertain their safety and efficacy by conducting follow up quantitative research, including trials, and focusing on specific innovations.

## Conclusion

COVID-19 and associated pandemic measures necessitated different approaches that resulted in service innovation, both technological as well as process innovations. From the perspective of service providers, some innovative solutions worked better than others in terms acceptability, accessibility, and safety for the providers as well as service users. The providers also noted that innovations implemented during COVID-19 pandemic and associated measures of control are emergency innovations and cautioned against fast and complete transitions, especially with regards telehealth, without an appropriate impact assessment. Additionally, another aspect of innovation sustainability that was questioned was “returning back normal” due to limited future funding that may result in a high innovation sunk cost. Providers were also critical of unequal innovation between different services that might ultimately not be contributing to providing high quality care to people who might be referred to multiple providers. Henceforth, hybrid solutions, service effectiveness assessment, and systemic investment in innovation across services were suggested to help to maintain and move innovation forward. The results of this study should help to understand the range of innovations that have emerged within mental health related helplines, support services and General Practices, understand the opportunities and challenges, as well as guide future research in this exciting area.

## Data availability statement

The original contributions presented in this study are included in the article/Supplementary material, further inquiries can be directed to the corresponding author.

## Ethics statement

The studies involving human participants were reviewed and approved by the University of Auckland Health Research Ethics Committee (AH3109). The patients/participants provided their written informed consent to participate in this study.

## Author contributions

SH and SF initiated the study. AP and BS conducted the qualitative data analyses. SF, SH, and KW supervised the drafting of the manuscript by AP. AP collected and prepared the data and interviewed the participants. All authors participated in the recruitment to the study and in active discussion in the process of writing of this study.

## Footnotes

1 A simplified referral process where communication between primary care clinicians and specialists/organisations is enhanced by enabling quick and secure referrals to be sent and received through an electronic platform, instead of a paper-based system where the referrals can be easily lost or misplaced (21).

2 A secure messaging channel for prescribing and dispensing systems to exchange prescription information electronically. It enables a prescription to be generated by the prescriber, transmitted to the NZePS health information exchange broker, and downloaded electronically at a community pharmacy (22).
