# Supplementary material for: COVID-19 related innovation in Aotearoa/New Zealand mental health helplines and telehealth providers – mapping solutions and discussing sustainability from the perspective of service providers
Source: Front Psychiatry. 2022 Aug 30;13:973261. doi: 10.3389/fpsyt.2022.973261 (PMC9468817; doi:10.3389/fpsyt.2022.973261)
Supplement: Supplementary file 1 [file Data_Sheet_1.pdf]

Appendix 1 – New Zealand COVID-19 Alert Levels Summary (10)

New Zealand COVID-19 **Alert Levels Summary**

Unite against COVID-19

- The Alert Levels are determined by the Government and specify the public health and social measures to be taken in the fight against COVID-19. Further guidance is available on **covid19.govt.nz**.
- The measures may be updated based on new scientific knowledge about COVID-19, information about the effectiveness of control measures in New Zealand and overseas, or the application of Alert Levels at different times (for example, the application may be different depending on if New Zealand is moving down or up Alert Levels).
- Different parts of the country may be at different Alert Levels. We can move up and down Alert Levels.
- Services including supermarkets, health services, emergency services, utilities and goods transport will continue to operate at any level. Employers in those sectors must continue to meet health and safety obligations.
- Restrictions are cumulative (for example, at Alert Level 4, all restrictions from Alert Levels 1, 2 and 3 apply).

Updated 27 August 2021

ELIMINATION STRATEGY – New Zealand is working together to eliminate COVID-19

Alert Level	Risk assessment	Measures that can be applied locally or nationally	
**Level 4 – Lockdown**<br>Likely the disease is not contained	• There is sustained and intensive community transmission.<br>• Outbreaks are widespread.	• Stay home in your bubble, other than for essential personal movement.<br>• Safe recreational activity is allowed in your local area.<br>• Travel is severely limited.<br>• All gatherings are cancelled, and all public facilities close.<br>• If you are working for an Alert Level 4 business or service and you have no available options for childcare, you can extend your household bubble to include a carer for your children.<br>• When leaving your home, you legally must wear a face covering:<br>◦ on public transport and at departure points, for example airports, train stations and bus stops<br>◦ if you are a driver or passenger of a taxi or ride-share vehicle<br>◦ when you visit a healthcare facility (other than for a patient)<br>◦ inside retail businesses that are still open, such as supermarkets, pharmacies and petrol stations.	• Businesses close except for essential services (for example, supermarkets, pharmacies, clinics, petrol stations) and lifeline utilities.<br>• Educational facilities close.<br>• Rationing of supplies and requisitioning of facilities is possible.<br>• Reprioritisation of healthcare services is possible.<br>• You are encouraged to wear a face covering whenever your leave the house.<br>• Green grocers, butchers, bakeries, and fishmongers can sell uncooked food items online. All orders legally must by delivery.
**Level 3 – Restrict**<br>Medium risk of community transmission – active but managed clusters	• There are multiple cases of community transmission.<br>• There are multiple active clusters in multiple regions.	• Stay home in your bubble, other than for essential personal movement, including going to work or school if you have to, or for local recreation.<br>• Keep 2 metres apart from people you do not know outside your bubble (including at supermarkets, petrol station and pharmacies), or 1 metre in controlled environments such as schools and workplaces.<br>• Stay within your immediate household bubble, but you can expand this to reconnect with close family/whānau, enable caregiving, or support isolated people. This extended bubble legally must remain exclusive.<br>• Schools (years 1 to 10) and Early Childhood Education centres can safely open, but with limited capacity. Children should learn at home if possible.<br>• You should work from home unless it is not possible.<br>• Businesses cannot have customers on site, unless it is a supermarket, bank, primary produce retailer, pharmacy, petrol station or hardware store providing goods to trade customers, or it is an emergency or critical situation.	• Other businesses can open premises, but customers cannot enter.<br>• Low-risk local recreation activities are allowed.<br>• Public facilities are closed (for example, libraries, museums, cinemas, food courts, gyms, pools, markets).<br>• Gatherings of up to 10 people are allowed but only for weddings, civil union ceremonies, funerals and tangihanga. Physical distancing and record keeping legally must be maintained.<br>• Healthcare services should use virtual, non-contact consultations where possible.<br>• Inter-regional travel is highly limited with limited permissions.<br>• People at high risk of severe illness, such as older people and those with existing medical conditions, are encouraged to stay at home where possible, and take additional precautions when leaving home. You may choose to work.<br>• You are encouraged to wear a face covering when leaving your home. You legally must wear a face covering:<br>◦ on public transport and at departure points, for example airports, train stations and bus stops<br>◦ if you are a delivery driver to residential addresses<br>◦ if you are a driver or passenger of a taxi or ride-share vehicle<br>◦ when you visit a healthcare facility (other than as a patient)<br>◦ inside businesses or services that are open, such as supermarkets, pharmacies and petrol stations.

**Level 2 – Reduce**<br>The disease is contained, but the risk of community transmission remains	• There could be limited community transmission.<br>• There are active clusters in more than one region.	• You can reconnect with friends and family, and socialise in groups of up to 100, go shopping and travel domestically, if following public health guidance.<br>• Keep 2 metres apart from people you don't know in retail stores. Try to keep 2 metres apart from people you don't know when out in public. Keep 1 metre apart in controlled environments like workplaces, where practicable.<br>• No more than 100 people allowed at social gatherings, including weddings, civil union ceremonies, birthdays, funerals and tangihanga.<br>• Businesses can open to the public if following public health guidance, including physical distancing and record keeping. Alternative ways of working encouraged where possible.<br>• Hospitality businesses legally must keep groups of customers separated and seated. Maximum of 100 people in a defined space.<br>• You legally must wear a face covering when:<br>◦ on public transport and at departure points, for example airports, train stations, bus stops, and in taxi or ride-share vehicles<br>◦ on flights<br>◦ you visit a healthcare facility (other than as a patient)<br>◦ you visit an aged care facility (other than as a patient)<br>◦ inside retail businesses, for example supermarkets, shopping malls, indoor marketplaces and takeaway food stores<br>◦ visiting the public areas within courts and tribunals, local and central Government agencies, and social service providers with customer service counters<br>• You legally must wear a face covering if you work:<br>◦ as a driver of a taxi or ride-share vehicle<br>◦ at close contact businesses, for example barbers, beauticians and hairdressers<br>◦ in a public facing role at a hospitality venue, for example a cafe, restaurant, bar or nightclub<br>◦ at retail businesses, such as supermarkets, shopping malls, indoor marketplaces, takeaway food stores<br>◦ in the public areas of courts and tribunals, local and central Government agencies, and social service providers with customer service counters<br>◦ at indoor public facilities, for example libraries and museums (but not swimming pools)	• Event facilities, including cinemas, stadiums, concert venues and casinos, can have more than 100 people at a time, provided that there are no more than 100 in a defined space, and the groups do not mix.<br>• Health and disability care services can operate as normally as possible.<br>• It is safe to send your children to schools, early learning services and tertiary education. There will be appropriate measures in place.<br>• People at higher risk of severe illness from COVID-19 (for example, those with underlying medical conditions, especially if not well-controlled, and older people) are encouraged to take additional precautions when leaving home. You may work, if you agree with your employer that you can do so safely.<br>• Passengers and workers in transport stations legally must keep 1 metre apart, as far as reasonably practicable.<br>• Sport and recreation activities are allowed, subject to conditions on gatherings, record keeping, and – where practical – physical distancing.<br>• Public facilities such as museums, libraries and pools can open if they comply with public health measures and ensure 1 metre physical distancing.
**Level 1 – Prepare**<br>The disease is contained in New Zealand	• COVID-19 is uncontrolled overseas.<br>• There could be sporadic imported cases.<br>• There could be isolated local transmission in New Zealand.	• There are border entry measures to minimise the risk of importing COVID-19 cases.<br>• Intensive testing for COVID-19 is carried out.<br>• Rapid contact tracing of any positive case is carried out.<br>• Schools and workplaces can open, and must operate safely.<br>• There are no restrictions on personal movement, but you are encouraged to maintain a record of where you have been.<br>• There are no restrictions on gatherings, but organisers are encouraged to maintain records to enable contact tracing.<br>• Recording keeping is a requirement at all Alert Levels at busy places and events so contact tracing can happen quickly and may help prevent future lockdowns. This requirement is for businesses and locations to take steps to ensure a record is kept. Places where it will be mandatory have systems and processes to ensure records are kept include:<br>• hospitality venues, including cafes, restaurants, bars, nightclubs (for customers)<br>• indoor event facilities, such as cinemas, theatres, casinos and concerts (for visitors only)<br>• aged care, healthcare facilities (for visitors only) • exercise facilities (for customers)<br>• close contact businesses, for example beauticians, barbers and hairdressers (for customers)<br>• indoor public facilities, such as libraries, museums and swimming pools (for visitors only)<br>• courts and tribunals, local and central government agencies, and social services providers with customer service counters (for visitors only)<br>• Social gatherings including those at marae, weddings, funerals, tangihanga and faith-based services.	• Stay home if you are sick, report flu-like or COVID-19 symptoms.<br>• Wash and dry your hands, cough into your elbow, do not touch your face.<br>• Avoid public transport or travel if you're sick.<br>• NZ COVID Tracer QR codes issued by the NZ Government legally must be displayed in workplaces and on public transport to enable use of the NZ COVID Tracer App for contact tracing.<br>• Face coverings are required on public transport and aircraft, but not inter-island ferries and school buses. Children under 12, passengers in taxis or ride-share services, and people with disabilities or mental health conditions do not have to wear face coverings.

Appendix 2A – Healthcare restrictions associated with national Alert Levels (11)

Alert Level	What does it mean for healthcare services
**Alert Level 4**	At Alert Level 4, you can only leave your house for essential personal movements, for example accessing healthcare like GPs, cancer services, disability and aged support services.<br><br>• Healthcare facilities will open and operate, where possible. You can access the treatments and medicines that you need to stay well.<br>• Minor health issues can become more serious if ignored and it is important that you have the treatments and medications you need. Do not delay seeking help for any health needs at any Alert Level.<br>• For other health services such as physiotherapists, dietitians and dentists, call to confirm whether appointments will be available in person, by phone or online.<br>• If you need medical assistance, phone your doctor or Healthline on 0800 611 116 first. You can travel to a healthcare facility, but it is recommended you call the facility before you go, so you know what to do when you get there. For example, you might need to use a certain entrance, or to wait outside in your car until called.<br>• Most consultations will happen over the phone or by videoconference to stop any risk of COVID-19 spreading by person-to-person contact.<br><br>**Getting routine care and repeat prescriptions**<br><br>• Call your doctor if you need a routine appointment or a repeat prescription for your regular medication. Your doctor can set up an appointment online or over the phone.<br>• Pharmacies will open, even in Alert Level 4. If you need to go to your local pharmacy for medication, call them first to make sure you can go instore.<br>• If you are an at-risk group you may want to ask someone to pick up your medications for you.<br><br>**Disability and aged care**<br><br>• At Alert Level 4, disability and aged care services will continue. Some home help may be available if it can be accessed safely.<br>• Carers are permitted to work at Alert Level 4. If you are a carer travelling to provide care or support for a person who is in a critical or terminal condition, we recommend you carry proof of your travel. This can include a letter from a medical professional confirming there is a person who needs urgent care or support and your destination.<br><br>**If you are due to give birth during Alert Level 4**

	• At Alert Level 4, health services will continue to operate. However, there may be some changes to the way you receive medical care if you are pregnant or have recently given birth. Contact your midwife or doctor if you have any concerns.<br>• When you give birth, maternity facilities may have restrictions on the number of visitors and support people you can have with you.
**Level 3**	At Alert Level 3, we need to restrict our movements. You should only leave your home for essential personal movement, such as accessing healthcare. Healthcare services will open and operate normally, where possible.<br><br>• Healthcare businesses, such as pharmacies, can open.<br>• Hospitals will remain open for emergency care. Some non-urgent services or treatment may be rescheduled.<br>• When visiting a healthcare facility at Alert Level 3, you must wear a face covering.<br>• Any appointments with a medical professional will need to be online or over the phone, where possible.<br>• Community health services and screening services can continue.<br>• Some disability and aged care services can continue but will be assessed on a case-by-case basis.
**Alert Level 2**	At Alert Level 2, where possible health and disability services will open and operate as normal.<br><br>• Emergency care is still available, but some appointments will move to online or over the phone where possible. Some non-urgent services or treatment may be rescheduled.<br>• Community health services and screening services will continue.<br>• Disability and aged care services continue under Alert Level 2, with extra consideration given to how at-risk people are.
**Alert Level 1**	Healthcare and disability services will run as normal at Alert Level 1.

Appendix 2B – Healthcare restrictions associated with national Alert Levels (12)

Alert Level	What does it mean for healthcare services
**Alert Level 4**	*Only urgent acute care* is conducted in person, maintaining public health guidelines. *Routine care is postponed*. If it's a medical emergency – call 111. (A medical emergency includes chest pain or tightness, difficulty breathing, choking, severe bleeding or bleeding that won't stop, sudden weakness or difficulty talking, fainting or unconsciousness). Emergency ambulance services continue to operate across New Zealand. For aged residential care, hospices and disability residential care facilities, family visits are not currently permitted. Pharmacies remain open. Healthline (0800 358 5453) has increased its workforce to handle increases in call volume.<br><br>Note: If you are unsure about what you can access in your area, please contact your local DHB or GP who can provide advice on appointments, visitor arrangements etc<br><br>**Hospitals**<br><br>• Hospitals operate in line with the National Hospital Response Framework<br>• Only urgent acute care is conducted in person, maintaining public health guidelines. Routine treatment or healthcare appointments are postponed.<br>• Elective surgery and non-essential outpatient hospital appointments are postponed<br>• Anyone who needs urgent medical attention – such as a medical emergency or going into labour – should follow the normal procedures.<br>• Cancer treatment will continue.<br>• Outpatient appointments continue but will be mainly online or over the phone.<br>• Visitors should check with the DHB first about whether visitors are allowed, as this may vary by ward in the hospital.<br><br>**Emergency ambulance services**<br><br>• Emergency ambulance services, including road ambulance and air ambulance, continue to operate throughout all COVID-19 alert levels.<br>• Emergency road ambulance services are provided by St John and Wellington Free Ambulance (for Wellington and the Wairarapa).<br>• All emergency ambulance providers adhere to strict infection prevention and control principles to help protect paramedics and patients from the risk of COVID-19.<br>• Please tell the call taker and paramedics if you may have been exposed to COVID-19. This will ensure that they able to use the appropriate PPE (eg, masks and gloves) to keep themselves and others safe while treating and transporting you.<br><br>**General practices**

	• General practices will be open, but appointments will still be conducted though telehealth or by phone where possible.<br>• It is important to still contact your health professional or Healthline (0800 611 116) as you normally would. When you call, your clinic will likely ask some questions to decide if an online, phone or in person appointment is required.<br>• Your doctor or nurse will continue to provide care for urgent issues, management of long-term conditions, mental health consultations, prescriptions of medication and the treatment of common illness. You can access all the treatments, vaccinations and medicines you need to stay well, whether or not the care you need relates to COVID-19. Specifically, childhood vaccinations can continue. Patients will be referred to specialists and for other treatment if needed.<br>• When you make an appointment that requires a visit in person, you will be asked questions around possible COVID exposure and symptoms, this is to keep you and others safe.<br><br>**Community health services**<br><br>• Primary and community health providers will operate in line with the Community Response Framework.<br>• Only urgent acute care is conducted in person, maintaining public health guidelines. Routine treatment or healthcare appointments are postponed.<br>• Community pharmacies remain open, but medicine management services will be provided over the phone where possible. Medicines may continue to be delivered to some people.<br>• Community midwives will provide services in a variety of ways, including face-to-face and on-line appointments.<br>• Community dental services will only provide urgent and emergency care which cannot be deferred or managed with medication. Routine care (non-essential and elective dentistry) will not be provided. Updates are provided on the Dental Council website.<br>• Appointments for allied health services such as physiotherapy, podiatry, optometry and Well Child Tamaraki Ora services will continue to be mainly online or over the phone.<br><br>**Screening services**<br><br>There are some changes to available health screening services offered. For details visit Time to screen<br><br>**Disability and aged care services**<br><br>• Disability and aged residential care are essential services so will continue.

	• Essential personal care services, such as toileting, washing and feeding, will be provided as usual.<br>• Some home help, such as house cleaning, may be available if it can be provided safely.<br>• Essential supported living services will continue.<br>• Planned respite services will be suspended, but urgent respite care may be provided.<br>• Support persons are permitted under right 8 of the Health and Disability Code.<br>• For aged residential care, hospices and disability residential care facilities, family visits are not currently permitted<br>• Family visits on compassionate grounds, such as in a palliative situation, will be considered on a case-by-case basis by the facility nursing staff, subject to public health advice, provider assessment and COVID-19 risk screening. Visits will be by appointment only. There is a limit to the number of designated visitors at one time.<br>• NGO delivered services, such as social connection, health promotion and education activities are suspended, however other non-contact ways of providing this support are encouraged, such as ZOOM or by phone.<br>• Retirement Villages operate within the same restrictions as the wider community, with on-site facilities closed.<br><br>**Mental health and addiction services**<br><br>• Inpatient and residential mental health and addiction services will operate as usual, although there may be fewer beds available, to reduce the possibility of transmission.<br>• Community mental health service appointments will be online or by phone where possible.<br>• Urgent and crisis community mental health services will continue as usual.<br>• There is a range of welfare, mental health and wellbeing programmes underway to provide support to New Zealanders. Read more: COVID-19: Mental health and wellbeing resources
**Level 3**	**Hospitals**<br><br>• Hospitals remain open for emergency care<br>• Some planned care, including elective surgery and radiology, will be provided.<br>• Some non-urgent services or treatment may be deferred.<br>• Outpatient appointments will continue but will be mainly online or over the phone.<br>• Visitors with no suspicion of COVID-19 will be allowed one-at-a-time, once a day. The number of visitors allowed per patient per day depends on where they are, and discretion may be applied on a case-by-case basis. Check with the DHB first.<br>• Women in labour in a maternity facility will be allowed one support partner, from her extended bubble for the duration of the labour and birth.

	**General practices**<br><br>• General practices will be open, but appointments will still be conducted online or by phone where possible. You can see your doctor or nurse face-to-face if required.<br>• Your doctor or nurse will continue to provide care for urgent issues, management of long-term conditions, mental health consultations, prescriptions of medication and the treatment of common illness.<br>• Patients will be referred to specialists and for other treatment if needed.<br>• It is important to still contact your health professional or Healthline (0800 611 116) as you normally would. You can access all the treatments, vaccinations and medicines you need to stay well, whether or not the care you need relates to COVID-19.<br>• Cervical and breast screening programmes will operate under Alert Level 3 at this stage.<br><br>**Community health services**<br><br>• Community pharmacies remain open, but medicine management services will be provided over the phone where possible. Medicines may continue to be delivered to some people.<br>• Community midwives will provide services in a variety of ways, including face-to-face and on-line appointments.<br>• Community dental services may provide face-to-face appointments for urgent or emergency care. Routine care (non-essential and elective dentistry) will not be provided. Updates are provided on the Dental Council website.<br>• Appointments for allied health services such as physiotherapy, podiatry, optometry and Well Child Tamaraki Ora services will continue to be mainly online or over the phone. Some face-to-face appointments may be provided for urgent appointments only, so long as professionals can take appropriate measures to manage public health risks.<br><br>**Screening services**<br><br>• Cancer screening programmes are continuing to operate, with appropriate safeguards in place to keep participants and staff safe. It is recommended that people over 70, or with pre-existing medical conditions, check with their health professional as to whether it is safe to attend appointments.<br>• Cervical and breast screening will continue for most women. The decision to screen those with existing medical conditions will be made on a case-by-case basis.<br>• Bowel screening invitations and home testing kits will continue to be sent out. If you receive a kit in the mail you should complete it and send it back as soon as possible.<br>• More information about bowel, breast and cervical screening can be found on the Time to Screen website.

	• Antenatal and newborn screening services will continue to be provided but with some changes to ensure the ongoing safety of women and babies. More information can be found on the National Screening Unit website.<br><br>**Disability and aged care services**<br><br>• Disability residential care will continue as usual.<br>• Any visitors will need to discuss with disability providers. Controlled visits with agreed and named family and whānau and close friends are allowed. A maximum of one visitor at any one time may visit the disabled person in their home.<br>• In aged residential care, only family visits for end of life / palliative care residents will be considered on a case-by-case basis.<br>• Family visits for residents in a hospice are allowed, but on a case-by-case basis, subject to public health direction and the hospice's assessment.<br>• Planned respite services will be suspended, but urgent respite care may be provided.<br>• Essential personal care services, such as toileting, washing and feeding, will be provided as usual.<br>• Some home help, such as house cleaning, may be available.<br><br>**Mental health and addiction services**<br><br>• Inpatient and residential mental health and addiction services will operate as usual, although there may be fewer beds available, to reduce the possibility of infection.<br>• Community mental health service appointments will be online or by phone where possible. There may be some face-to-face appointments.<br>• Urgent and crisis community mental health services will continue as usual.<br>• There is a range of welfare, mental health and wellbeing programmes underway to provide support to New Zealanders.
**Level 2**	**Hospitals**<br><br>• Hospitals remain open for emergency care, whether or not that care relates to COVID-19.<br>• Planned care, including elective surgery and radiology, will be provided in order of clinical priority.<br>• Some non-urgent services or treatment may be deferred.<br>• Outpatient appointments will continue but will be mainly online or over the phone.<br>• Visitors with no suspicion of COVID-19 will be allowed to visit hospitals. The number of visitors allowed per patient per day depends on where they are, and discretion may be applied on a case-by-case basis. Check with the DHB first. More information is available in the Questions and answers.

- Women in labour in a maternity facility will be allowed one support partner for the duration of the labour and birth.

General practices

- General practices will be open, but appointments will still be conducted online or by phone where possible. You can see your doctor or nurse face-to-face if required.
- Your doctor or nurse will continue to provide care for urgent issues, management of long-term conditions, mental health consultations, prescriptions of medication and the treatment of common illness.
- Patients will be referred to specialists and for other treatment if needed.
- It is important to still contact your health professional or Healthline (0800 611 116) as you normally would. You can access all the treatments, vaccinations and medicines you need to stay well, whether or not the care you need relates to COVID-19.
- All usual primary care continues including screening, acute care, long-term condition support, preventative services, antenatal and newborn care, and routine health needs such as mental health consults, prescription of contraception and other medication and treatment of common illnesses.

Community health services

- Community pharmacies remain open and all pharmacy services continue – including dispensing, medicines advice, vaccinations, community pharmacy warfarin monitoring service, long term conditions services and provision of medicines related health information. Some services will use online/phone, with in-person services available for people who do not have reliable access to technology and the internet.
- Community midwives will provide services in a variety of ways, including face-to-face and on-line appointments.
- Community dental services are open for routine, urgent and emergency care for people without COVID-19 or who those who are symptomatic. Confirmed and probable cases of COVID-19, close contacts of confirmed or probable cases of COVID-19 and people waiting for test results, can receive urgent and emergency dental treatment with clinicians meeting PPE and room requirements. Updates are provided on the Dental Council website.
- Appointments for allied health services such as physiotherapy, podiatry, optometry and Well Child Tamaraki Ora services can operate as normal after an appropriate COVID-19 risk screen, however for most people these will continue to be mainly online or over the phone.
- Māori and community health providers
- Māori and community health providers are open at Alert Level 2. They can operate as normal after an appropriate COVID-19 risk screen, however for most people appointments will continue to be mainly online or over the phone.

- Providers contracted to do so will continue to provide community-based and mobile testing for COVID-19.

Screening services

- Cancer screening programmes are continuing to operate, with appropriate safeguards in place to keep participants and staff safe. It is recommended that people over 70, or with pre-existing medical conditions, check with their health professional as to whether it is safe to attend appointments.
- Cervical and breast screening will continue for most women. The decision to screen those with existing medical conditions will be made on a case-by-case basis.
- Bowel screening invitations and home testing kits will continue to be sent out. If you receive a kit in the mail you should complete it and send it back as soon as possible.
- More information about bowel, breast and cervical screening can be found on the Time to Screen website.
- Antenatal and newborn screening services will continue to be provided but with some changes to ensure the ongoing safety of women and babies. More information can be found on the National Screening Unit website.

Disability and aged care services

- Disability residential care continues under all alert levels. All services will follow COVID-19 risk screening and infection prevention control, physical distancing measures, and record people’s details to enable contact tracing. PPE guidance is to be followed.
- Extra consideration will be given to how at-risk resident’s health will be protected.
- Level 2 also allows limited opening of facility-based respite services for disabled people. Facilities will contact disabled people and families and whānau to let them know how they will operate following Alert Level 2 rules. Flexibility for respite paid for under Individualised Funding remains under Alert Level 2.
- Essential personal care services, such as toileting, washing and feeding, will be provided as usual.
- Home based personal care services, such as showering and feeding, and home help, such as cleaning, are available. Infection prevention and control measures must be adhered to for essential care services that require close physical contact. Staff movement should be minimised between homes and household management activities should maintain physical distancing where possible. All equipment and modification services are now available following Alert Level 2 rules.

Mental health and addiction services

	• Inpatient and residential mental health and addiction services will operate as usual, although there may be fewer beds available, to reduce the possibility of infection.<br>• Community mental health service appointments will be online or by phone where possible. There may be some face-to-face appointments.<br>• Urgent and crisis community mental health services will continue as usual.<br>• There is a range of welfare, mental health and wellbeing programmes underway to provide support to New Zealanders.<br>• Visitors with no suspicion of COVID-19 will be allowed one-at-a-time, once a day. The number of visitors allowed per patient per day depends on where they are, and discretion may be applied on a case-by-case basis. Check with the DHB first.
**Alert Level 1**	Health and disability services will be running as normal under Alert Level 1 but some precautions will be in place to protect people who are more at risk of severe COVID-19 from exposure to the virus.<br><br>Strict adherence to infection prevention and control protocols will continue, and staff who are unwell must stay at home.<br><br>COVID-19 related precautions are not expected to affect the availability or timeliness of health or disability services.<br><br>Strict adherence to infection prevention and control protocols and COVID-19 risk screening for patients may be a requirement prior to arrival, or on entrance to health facilities. Staff may ask you if you have been overseas recently, had contact with anyone who has been overseas recently, or had any potential recent exposure to COVID-19 such as being a close contact of a confirmed or probable case. People with acute respiratory symptoms might be asked to access services digitally, or over the phone, rather than in person. You may also be asked to wear a face mask to protect others while awaiting assessment.<br><br>COVID-19 testing will be done in places including Community Based Assessment Centres (CBACs), or general practices.<br><br>**Hospitals**<br><br>Hospitals are open as usual and will be using all available additional capacity to address back-logs of people whose care was delayed during Alert Levels 2, 3 and 4. Backlogs for outpatient appointments, diagnostic services and planned care, including elective surgery and radiology, will be addressed in order of clinical priority. Infection prevention and control measures are an ongoing priority to ensure the safety of patients, visitors and staff from all infections. These could include the use of Personal Protective Equipment (PPE) depending on the nature of interactions and care required. Outpatient appointments will continue to be delivered via phone, video-call or other remote technology where

	clinically appropriate and suitable for patients. Visitors are important for patients in hospital, but overcrowding must be avoided, and we must continue to keep our patients, health workers and visitors safe, so each DHB will determine appropriate visiting arrangements for their different clinical areas, within guidance provided by the Ministry of Health.<br><br>**General practices**<br><br>General practices will be open and will offer the full range of services that they delivered prior to COVID-19. Patients should have flexibility when it comes to accessing their care and will continue to be offered both telehealth or in person consultations. In-person visits may be preceded by a telephone call assessment, or assessment at the entrance to the practice to assess the patient's risk of COVID-19. Infection prevention and control measures are an ongoing priority to ensure the safety of patients, visitors and staff from all infections. These could include the use of Personal Protective Equipment (PPE) depending on the nature of care required. COVID-19 testing, aligned to guidance from the Ministry of Health, can be done in several places including Community Based Assessment Centres (CBACs), or general practices.<br><br>**Community health services**<br><br>Community based health services generally return to normal, with infection prevention and control measures and other public health measures to prevent the spread infections. These include: hand hygiene, respiratory hygiene and cough etiquette, regular cleaning of high touch surfaces and items as well as the use of Personal Protective Equipment (PPE) where appropriate and according to risk assessments. Contract tracing will be enabled, for example, by using the NZCOVID Tracer app. Staff who are unwell must stay home and not go to work.<br><br>*Allied health services*<br><br>Appointments for allied health services such as physiotherapy, podiatry, and optometry can operate as normal after COVID-19 risk screening. Patients should have flexibility when it comes to accessing their care and will continue to be offered both telehealth or in person consultations. In-person visits may be preceded by a telephone call or assessment at reception to assess the patient's risk of COVID-19, according to Ministry of Health guidance.<br><br>*Community pharmacies*<br><br>Community pharmacies will offer the full range of their services, and appointments will resume as usual. Customers with acute respiratory symptoms will be asked not to go to the pharmacy where possible and to phone for advice first.<br><br>*Community midwives*

	Community midwives will resume care as normal. Individual DHBs will determine the visiting/support policy for their maternity units. However, hospitals are encouraged to allow birthing women the same number of support people as before the COVID-19 response.<br><br>*Community dental services*<br><br>Community dental services are open for routine, urgent and emergency care. Confirmed and probable cases of COVID-19, and people waiting for COVID-19 test results, can receive urgent and emergency dental treatment only with clinicians meeting PPE and room requirements. Updates are provided on the Dental Council website. Scheduling of those with medical conditions increasing the risk of severe illness from COVID-19, or of those over 70 will be carefully managed.<br><br>*Well Child Tamariki Ora services*<br><br>Well Child Tamariki Ora services return to normal with emphasis on hygiene measures and assessing parents and children for COVID-19 risk before contact with their provider. As services return to normal Well Child Tamariki Ora providers will prioritise vulnerable whānau and those with young pēpi who have missed care during higher Alert Levels.Virtual care delivery will continue to be used where it is appropriate (e.g. for extra support or assessment).<br><br>**Screening services**<br><br>All screening services return to normal operation under Alert Level 1, however the facilities where these services take place may have some additional COVID-19 precautions (such as basic COVID-19 risk assessments) in place. You should contact the service provider ahead of time to discuss this. Basic hygiene measures including hand hygiene, respiratory hygiene and cough etiquette, as well as cleaning of surfaces and equipment will continue. More information about bowel, breast and cervical screening can be found at Time to Screen.<br><br>*Cancer screening*<br><br>Cancer screening programmes are now operating as normal. People who missed their breast or cervical screening appointments during Alert Levels 2, 3 or 4 will be contacted to make new appointments. Your provider will be in contact.<br><br>*Bowel screening*<br><br>Bowel screening is now operating as normal. People who received a home test kit and were asked to put it aside until further advice, can complete it and send it back. If you have any concerns about your symptoms, please talk to your GP.DHBs are scheduling colonoscopies and new invitations are now going out to patients.

Antenatal and newborn screening

Antenatal and newborn screening services are returning to being provided as part of routine maternity care. Please contact your maternity care provider for advice about any difference in procedures for attending appointments during Alert Level 1. More information about maternity services

Immunisations

Immunisations are being provided as normal in Alert Level 1, however the facilities or providers who conduct these services may have some additional COVID-19 precautions in place. People who have missed immunisations during Alert Levels 4, 3 and 2 will be contacted to schedule an appointment.

If you need to organise an immunisation it is best to contact your healthcare provider ahead of time so they can explain any different processes that are in place during Alert Level 1.Basic hygiene measures including hand hygiene, respiratory hygiene and cough etiquette, as well as cleaning of surfaces and equipment will continue.

Disability and aged care services

All Disability Support Services will operate as normal under Alert Level 1 with visiting policies as they were before the COVID-19 Alert Level system. All services will have COVID-19 risk screening, will adhere to infection prevention controls, and will record people's details to enable contact tracing where appropriate. Alert Level 1 PPE guidance is to be followed. People who are more at risk of becoming very unwell from COVID-19 may choose to take extra precautions. These people may want to consider discussing with their doctor what kind of precautions might be appropriate in their situation. You can find more information about disability support services that are available under Alert Level 1 at COVID-19: Information for disabled people and their family and whānau.

Aged residential care

Aged residential care will operate as normal under Alert Level 1 with visiting policies operating as they were before the COVID-19 Alert Level system. 14-day isolation for admissions to aged residential care is not required. Facilities will have COVID-19 risk screening, and adhere to Alert Level 1 public health measures. A record of where people have been are encouraged to be kept for contact tracing purposes, in particular where visitors are not routine (i.e. not direct family / regular visitors). All planned and urgent respite care previously provided in aged residential care is available.

Home based support services

	Home based support services, including personal cares (e.g. showering and feeding), and household management (e.g., cleaning) will operate as normal and will continue to adhere to infection prevention control measures.<br><br>*Respite, Carer Support, and day services*<br><br>Respite, Carer Support, and day services are all operating as normal under Alert Level 1.<br><br>Respite care and day services (and transport options to and from day services) will adhere to infection prevention control measures and will record peoples details to enable contact tracing purposes.<br><br>People shouldn't be provided respite care in the community, take part in health promotion activities or be provided other community supports if they are unwell.<br><br>Day services will continue to provide remote support for those that require it.
**All Alert Levels**	**Precautions in healthcare facilities**<br><br>At all Alert Levels healthcare facilities and businesses will take precautions against the risk of COVID-19 transmission. These can include:<br><br>• risk screening on arrival<br>• contact tracing<br>• providing services over the phone or online if you have any symptoms<br>• asking you to wear a face covering while visiting the healthcare facility.<br><br>When accessing healthcare, you must take precautions against the spread of COVID-19, including:<br><br>• physical distancing<br>• wash or sanitise your hands<br>• keep track of where you have been<br>• wear a face covering, where possible

Appendix 3 – Interview schedule

Service delivery changes

1. How did the situation with COVID-19 affected the service delivery?

2. What were the main challenges (e.g. new technologies, capacity issues, lack of prior experience and training in the setting of a pandemic)?
3. What are the changes that your organisation had to make? Was it difficult/easy?
4. What worked well/not so well?
5. How do you think it affected service effectiveness? Were there any differences in general communication patterns (i.e. maintenance of anonymity, levels of disclosure)?
6. What have you learned from it? Would you do anything differently?
7. What service changes do you see as useful?
8. What changes will you maintain (even outside of COVID-19)?

Contextual information

1. Tell us a little bit more about your organisation
2. Tell us a little bit more about yourself/your role in it?
3. How many colleagues do you have? How many people do you manage? Who do you report to?
4. What does your work mean for you?
5. How have the recent events affected your job?
6. How do you think your colleagues handling this? Your reportees/ your management?
7. What organisational challenges you personally have experienced?
8. Do you feel the same about your job/role as before? What have changed?
9. Do you have a say in strategic decision? If you would have a say – what would you have done differently?

References

1. New Zealand Ministry of Health. History of the COVID-19 Alert System [Internet]: Ministry of Health; 2021. Available from: https://covid19.govt.nz/alert-levels-and-updates/history-of-the-covid-19-alert-system/.
2. BBC. Christchurch shootings: 49 dead in New Zealand mosque attacks [Internet]: BBC; 2019. Available from: https://www.bbc.com/news/world-asia-47578798.
3. The Guradian. New Zealand cancels Christchurch attacks memorial due to coronavirus fears [Internet]: The Guradian; 2020. Available from: https://www.theguardian.com/world/2020/mar/14/new-zealand-cancels-christchurch-attacks-memorial-due-to-coronavirus-fears.
4. Radio New Zealand. Thousands of NZers march for Black Lives Matter [Internet]: Radio New Zealand; 2020. Available from: https://www.rnz.co.nz/news/national/418971/thousands-of-nzers-march-for-black-lives-matter.
5. Radio New Zealand. As it happened: Police officer shot dead in West Auckland [Internet]: Radio New Zealand; 2020. Available from: https://www.rnz.co.nz/news/national/419381/as-it-happened-police-officer-shot-dead-in-west-auckland.
6. New Zealand Parliament. Looking back at delayed elections in New Zealand's history [Internet]: New Zealand Palaiment; 2020. Available from:

https://www.parliament.nz/en/get-involved/features/looking-back-at-delayed-elections-in-new-zealands-history/.
7. Electoral Commission. Media release: Election day will be 17 October [Internet]: Electoral Commission; 2020. Available from: https://elections.nz/media-and-news/2020/election-day-will-be-17-october/.
8. Public Holidays Global. New Zealand Public Holidays 2020 [Internet]: Public Holidays Global; 2020. Available from: https://publicholidays.co.nz/2020-dates/.
9. Roy EA. New Zealand partially shuts central Auckland over mystery Covid case Queenstown [Internet]: The Guradian; 2020. Available from: https://www.theguardian.com/world/2020/nov/12/new-zealand-investigates-mystery-covid-case-in-auckland.
10. New Zealand Government. Summary table of COVID-19 Alert Levels 2021 [Internet] [27.08.2021]: Available from: https://covid19.govt.nz/alert-levels-and-updates/legislation-and-key-documents/#key-documents.
11. New Zealand Government. How to access healthcare and emergency services 2021. Available from: https://covid19.govt.nz/health-and-wellbeing/how-to-access-healthcare-and-emergency-services/#healthcare-at-alert-level-3.
12. New Zealand Ministry of Health. Health and disability services at different Alert Levels 2021. Available from: https://www.health.govt.nz/our-work/diseases-and-conditions/covid-19-novel-coronavirus/covid-19-health-advice-public/health-and-disability-services-different-alert-levels.
